# Supplementary material for: Carbon Quantum Dots Prepared with Chitosan for Synthesis of CQDs/AuNPs for Iodine Ions Detection
Source: Nanomaterials (Basel). 2018 Dec 13;8(12):1043. doi: 10.3390/nano8121043 (PMC6315431; doi:10.3390/nano8121043)
Supplement: Supplementary file 1 [file nanomaterials-08-01043-s001.pdf]

Supplementary Information

Carbon Quantum Dots Prepared with Chitosan for Synthesis of CQDs/AuNPs for Iodine Ions Detection

Juanjuan Song [1,†], Li Zhao [1,†], Yesheng Wang [1], Yun Xue [1], Yujia Deng [1], Xihui Zhao [1,2,*] and Qun Li [1,2]

[1] School of Chemistry and Chemical Engineering, Qingdao University, Qingdao 266071, China; songjuan2017@163.com (J.S.); 2017020829@qdu.edu.cn (L.Z.); 2016020405@qdu.edu.cn (Y.W.); xueyun@qdu.edu.cn (Y.X.); dengyujia@qdu.edu.cn (Y.D.); qunli@qdu.edu.cn (Q.L.)

[2] State Key Laboratory of Bio-Fibers and Eco-Textiles, Shandong Collaborative Innovation Center of Marine Biobased Fibers and Ecological Textiles, Institute of Marine Biobased Materials, Qingdao University, Qingdao 266071, China

* Correspondence: zhaoxihui@qdu.edu.cn; Tel.: +86-0532-85955529

† These authors contributed equally to this work.

Test method for deacetylation degree of chitosan:

The degree of deacetylation (DD) of chitosan was determined by titration as follows:

0.20 g vacuum-dried sample was dispersed in 20 mL, 0.1 mol/L HCl and then titrated with 0.1 mol/L NaOH. The pH value and corresponding volume of NaOH solution was recorded and a titration curve was plotted. This plot had two inflection points, where the corresponding volumes were recorded as V1 and V2. The DD value was calculated according to the following equation

$$\text{Degree of deacetylation (DD)} = ((V2-V1)\times c\times 0.016)/(0.094*W) \quad (1)$$

Where c is the concentration of NaOH solution, and W is the sample weight (g).

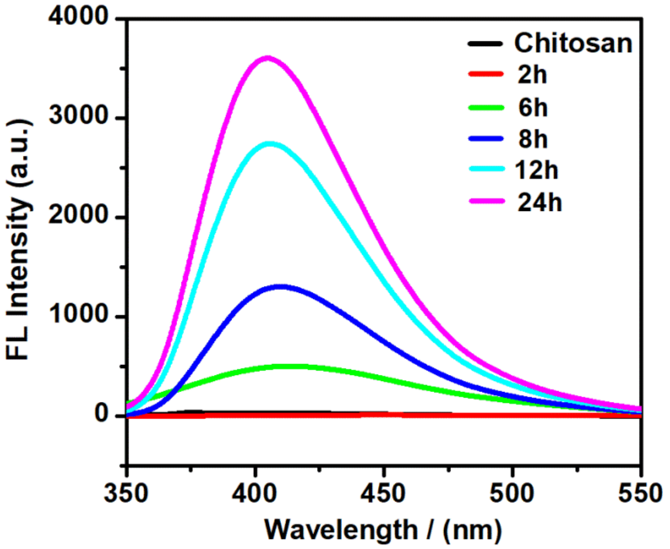

Figure S1 Fluorescence emission spectra of CQDs obtained by chitosan hydrothermal carbonization with different times (at a maximum excitation wavelength of 330 nm).

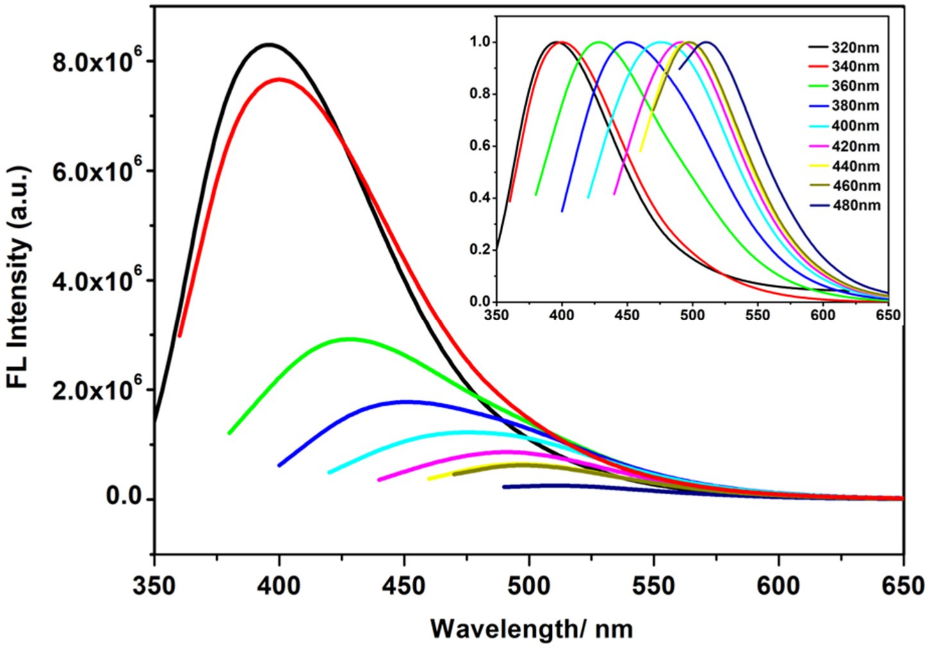

Figure S2 Fluorescence emission spectra of the CQDs obtained under excitations from 280 to 480 nm with 20 nm interval. Inset: Normalized Fluorescence emission spectra.

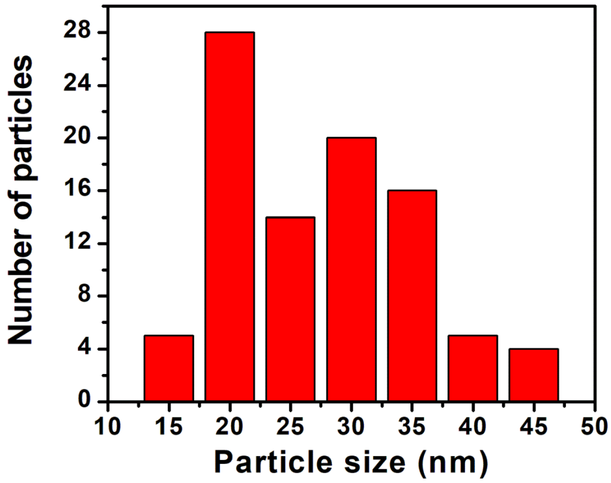

Figure S3 size distribution histogram of CQDs/AuNPs.

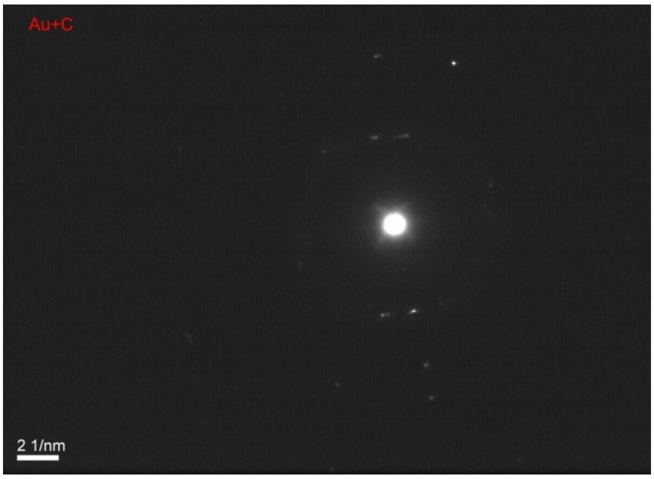

Figure S4 SAED of CQDS/AuNPs.

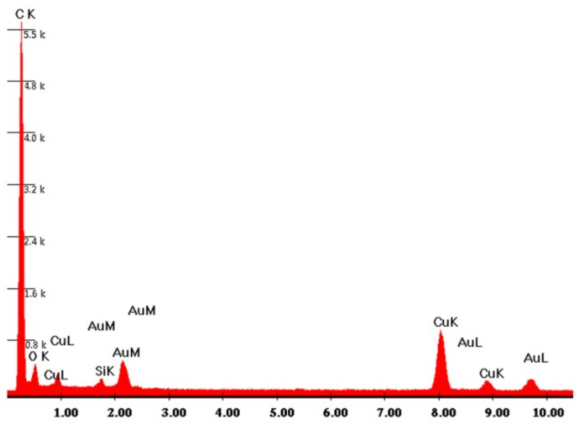

Figure S5 Energy dispersive X-ray spectrum (EDS) of the CQDS/AuNPs showing the peaks for C, O, Cu and Au.

Table S1 Results for the determination of iodide in salt samples (n=3).

Sample	Spiked (μM)	Measured (μM)	Recovery (%)
salt	0	-	-
	5	4.92	98.4
	10	10.74	107.4
	20	21.10	105.5

Table S2 Comparison of the proposed iodide detection method with other reported methods.

Methods	Probe	Linear range/(M)	LOD/(M)	Ref.
Colorimetric	ATTP-AuNPs	5.0×10^{-7} to 6.0×10^{-6}	1.5×10^{-8}	[2]
Fluorescence	N-CQDs	1×10^{-4} to 2.0×10^{-3}	1.0×10^{-5}	[17]
Colorimetric	Cu@Au Nanoparticles	—	5.0×10^{-6}	[S1]
Colorimetric	FCD	—	4.3×10^{-7}	[S2]
Fluorescence Turn off and colorimetric	$C_{20}H_{20}N_4O_5$	6.25×10^{-6} to 3.12×10^{-5}	3×10^{-8}	[S3]
Colorimetric	$C_{22}H_{22}N_4O_6S_2$	—	5.31×10^{-6}	[S4]
Fluorescence	FITC-Au nanoparticles	1.0×10^{-7}–1.0×10^{-6}	5×10^{-8}	[S5]
Colorimetric	silver nanoparticles	6.0×10^{-6}–8.0×10^{-6}	2.5×10^{-4}	[S6]
Colorimetric	CQDs/AuNPs	2.0×10^{-5}-4.0×10^{-4}	2.3×10^{-6}	This work

References

S1. Zhang, J.; Xu, X.; Yang, C.; Yang, F.; Yang, X. Colorimetric iodide recognition and sensing by citrate-stabilized Core/shell Cu@Au nanoparticles. *Anal. Chem.* **2011**, *83*, 3911–3917.

S2. Jafari, M.; Badiei, A.; Shayesteh, A.; Amiri, A. Fluorescent and colorimetric dual mode probe for detection of iodide through iodide/triiodide equilibrium. *J. Photochem. Photobiol. A Chem.* **2018**, *364*, 336–343.

S3. Thakur, A.; Bhatta, S. R.; Mondal, B.; Kakash, D.; Chawla, P. Naphthalene-glycine conjugate: An extremely selective colorimetric chemosensor for iodide ion in aqueous solution. *Sensors Actuators, B Chem.* **2018**, *267*, 617–626.

S4. Goh, H.; Nam, T. K.; Singh, A.; Singh, N.; Jang, D. O. Dipodal colorimetric sensor for Ag^+ and its resultant complex for iodide sensing using a cation displacement approach in water. *Tetrahedron Lett.* **2017**, *58*, 1040–1045.

S5. Wei, S. C.; Hsu, P. H.; Lee, Y. F.; Lin, Y. W.; Huang, C. C. Selective detection of iodide and cyanide anions using gold-nanoparticle-based fluorescent probes. *ACS Appl. Mater. Interfaces* **2012**, *4*, 2652–2658.

S6. Kumar, A.; Chhatra, R. K.; Pandey, P. S. Synthesis of click bile acid polymers and their application in stabilization of silver nanoparticles showing iodide sensing property. *Org. Lett.* **2010**, *12*, 24–27.
